# TGF-β Signaling Loop in Pancreatic Ductal Adenocarcinoma Activates Fibroblasts and Increases Tumor Cell Aggressiveness

**DOI:** 10.3390/cancers16213705

**Published:** 2024-11-01

**Authors:** Noemi di Miceli, Chiara Baioni, Linda Barbieri, Davide Danielli, Emiliano Sala, Lucia Salvioni, Stefania Garbujo, Miriam Colombo, Davide Prosperi, Metello Innocenti, Luisa Fiandra

**Affiliations:** Department of Biotechnology and Biosciences, University of Milano-Bicocca, Piazza Della Scienza 2, 20126 Milan, Italy; dimicelinoemi@gmail.com (N.d.M.); c.baioni1@campus.unimib.it (C.B.); linda.barbieri@unimib.it (L.B.);d.danielli@campus.unimib.it (D.D.); salaemiliano@gmail.com (E.S.); lucia.salvioni@unimib.it (L.S.); stefania.garbujo@unimib.it (S.G.); miriam.colombo@unimib.it (M.C.); davide.prosperi@unimib.it (D.P.)

**Keywords:** tumor microenvironment, pancreatic ductal adenocarcinoma, CAF–cancer cell crosstalk, TGF-β, transwell, spheroids

## Abstract

Background: The interaction between cancer cells and cancer-associated fibroblasts (CAFs) is a key determinant of the rapid progression, high invasiveness, and chemoresistance of aggressive desmoplastic cancers such as pancreatic ductal adenocarcinoma (PDAC). Tumor cells are known to reprogram fibroblasts into CAFs by secreting transforming growth factor beta (TGF-β), amongst other cytokines. In turn, CAFs produce soluble factors that promote tumor-cell invasiveness and chemoresistance, including TGF-β itself, which has a major role in myofibroblastic CAFs. Such a high level of complexity has hampered progress toward a clear view of the TGFβ signaling loop between stromal fibroblasts and PDAC cells. Methods: Here, we tackled this issue by using co-culture settings that allow paracrine signaling alone (transwell systems) or paracrine and contact-mediated signaling (3D spheroids). Results: We found that TGF-β is critically involved in the activation of normal human fibroblasts into alpha-smooth muscle actin (α-SMA)-positive CAFs. The TGF-β released by CAFs accounted for the enhanced proliferation and resistance to gemcitabine of PDAC cells. This was accompanied by a partial epithelial-to-mesenchymal transition in PDAC cells, with no increase in their migratory abilities. Nevertheless, 3D heterospheroids comprising PDAC cells and fibroblasts allowed monitoring the pro-invasive effects of CAFs on cancer cells, possibly due to combined paracrine and physical contact-mediated signals. Conclusions: We conclude that TGF-β is only one of the players that mediates the communication between PDAC cells and fibroblasts and controls the acquisition of aggressive phenotypes. Hence, these advanced in vitro models may be exploited to further investigate these events and to design innovative anti-PDAC therapies.

## Simple Summary

In pancreatic ductal adenocarcinoma (PDAC), the interaction between tumor cells and the tumor microenvironment is critical in regulating cancer progression. In particular, cancer-associated fibroblasts (CAFs) affect the invasive activity and resistance to chemotherapy of tumor cells by means of contact-mediated and paracrine signals, the latter including transforming growth factor beta (TGF-β). By using advanced transwell and spheroid co-culture models as exploratory tools, we showed while that TGF-β is involved in the interplay between PDAC cells and fibroblasts and promotes the acquisition of aggressive phenotypes, additional as-yet-unknown factors play a role. We propose that these advanced cell culture models provide a pathologically relevant tractable system to investigate the role of the tumor microenvironment in PDAC progression and develop novel treatment strategies.

## 1. Introduction

Pancreatic ductal adenocarcinoma (PDAC) is the most common type of pancreatic neoplasm, accounting for approximately 85% of all pancreatic tumors. It is one of the deadliest tumors, with a 5-year survival rate of around 13% (in the United States, 2013–2019) [1]. Diagnosed cases are steadily increasing and forecasts say that PDAC will become the second leading cause of cancer-related death by 2030 [2]. PDAC is accompanied by desmoplasia in and around the tumor, which leads to very peculiar masses in which tumor cells represent less than 20% of the total volume, the remaining 80% being the desmoplastic stroma [3]. The tumor microenvironment (TME) of the PDAC has a key role as it regulates the growth, invasion, immune evasion, and drug resistance of tumor cells, also acting as a physical barrier against administered treatments. The cellular components of the TME, including endothelial cells, immune cells, and cancer-associated fibroblasts (CAFs), create a unique system of cell–cell interactions and produce soluble mediators (e.g., growth factors, metalloproteases, cytokines) [4] that can regulate a complex signaling network promoting tumor progression and chemoresistance. Among these soluble mediators, transforming growth factor beta (TGF-β) plays a key role: the TGF-β released into the TME by cancer cells and other cell types [5,6,7] is responsible for differentiation of CAFs, which are able to produce this cytokine as well [6,8,9]. Thus, CAFs contribute to the high TGF-β levels in the TME, which are involved in conferring hyperproliferative, metastatic, and chemoresistant phenotypes on tumor cells [10,11].

In PDAC, CAFs are very heterogeneous and comprise several, often coexisting, subpopulations that differ in terms of function, localization, and expression of molecular markers [12]. TGF-β is able to induce resident fibroblasts to become myofibroblast-like CAFs (myCAFs), which show high α-SMA expression, stiffness, and cell elongation [13,14].

TGF-β is known to induce the desmoplastic reaction in the TME of PDAC [15], which impairs the delivery of therapeutics at the tumor site by tightening the vasculature and increasing the interstitial fluid pressure [16].

TGF-β also induces epithelial-to-mesenchymal transition (EMT) of PDAC cells [17] through both SMAD-dependent and -independent pathways, resulting in the metastatic dissemination of cancer cells [18]. Furthermore, TGF-β is involved in PDAC chemoresistance, to which the hyper-desmoplastic texture of the stroma and its altered vascularization indirectly contribute by limiting drug penetration into the tumor mass. Moreover, TGFβ-activated CAFs release exosomes containing miRNAs that may increase the expression of the chemoresistance-inducing factor Snail [19,20]. Direct effects of TGFβ on cancer cells’ chemoresistance are mediated by the TGF-β/SMAD pathway and miRNAs targeting components of the TGF-β pathway (SMAD2, SMAD3, SMAD4). The resistance to chemotherapeutic drugs is due to the activation of alternative survival or anti-apoptotic signaling pathways or to increased expression of ABC multi-drug transporters facilitating drug efflux [21]. In PDAC, CAF-released TGF-β has been demonstrated to contribute to cancer progression and gemcitabine resistance by upregulating the expression of ATF4 via the SMAD2/3 axis [22].

As the role of TGF-β as a tumor promoter of PDAC is well documented, multiple studies have characterized its downstream pathways and activities, in order to identify possible new therapeutic approaches [18]. Nevertheless, despite the many available studies reporting TGF-β involvement in CAF activation and PDAC progression and chemoresistance, the role of TGF-β in early PDAC remains controversial, as there is evidence for both tumor-promoting and suppressor activity [23,24,25]. This is partly due to the fact that studying the TGF-β signaling loop is challenging. Here, we leveraged advanced co-culture systems consisting of human PANC-1 or MIA-Paca2 tumor cells and human lung fibroblast MRC-5, which retain many features of pancreatic stromal cells [26], including the ability to enhance the invasiveness of pancreatic cancer cells [27,28,29,30], to bypass this problem. We investigated the effects of the TGF-β released by PDAC cells on fibroblasts’ activation, and those exerted by the TGF-β released by activated fibroblasts on PDAC proliferation, invasiveness, and chemoresistance. We report that a TGF-β signaling loop contributes to inducing the activation of fibroblasts and enhances the aggressiveness of PDAC cells.

## 2. Materials and Methods

### 2.1. Cell Culture

Human PANC-1 cell line and MRC-5 were obtained from the American Type Culture Collection (distributed by LGC Standards, Milan, Italy). MIA-Paca2 cells were kindly provided by the Laboratory of Prof. Chiaradonna (University of Milano-Bicocca, Milan, Italy). All cell lines were cultured in High Glucose Dulbecco’s Modified Eagle Medium (DMEM) with 1% penicillin/streptomycin (P/S), 1% L-glutamine, and 10% fetal bovine serum (FBS). Cells were grown at 37 °C in 5% CO_2_ and under controlled humidity. Cell culture chemicals and reagents were bought from Euroclone (Milan, Italy). Cell growth and health were observed daily with a Paula digital microscope (Leica, Milan, Italy).

### 2.2. Reagents for Cell Treatment

Transforming growth factor TGF-β (ab50036, Abcam, Cambridge, UK) was maintained at −20 °C in 2 mg/mL PBS-BSA. The TGF-β receptor 1 (TGF-βR1) inhibitor SB431542 (S1067—10 mg, Selleck, by Aurogene, Rome, Italy) and the SMAD3 inhibitor SIS3 (A16065—5 mg, Adooq Bioscience, by CliniSciences, Rome, Italy) were kept at −80 °C and diluted in sterile 100% ethanol solvent. Gemcitabine was purchased from Merk Life Science S.r.l (Milan, Italy) and diluted in water.

### 2.3. Transwell Mono- and Co-Cultures

Transwell mono- and co-cultures were performed as already described in our previous work on a murine breast cancer model [31]. Human PANC-1 or MIA-Paca-2 cells were trypsinized and seeded in a 12-well plate (30,000 cells/well) in 1.5 mL of DMEM. Human MRC-5 fibroblasts were trypsinized and seeded on 12-well Cell Culture Inserts, with a membrane of 0.4 µm pore size (ThinCert^®^—Greiner Bio-One, by Euroclone, Milan, Italy) (30,000 cells/insert). In the co-culture condition, the inserts bearing the MRC-5 were moved into the multiwell with the tumor cells after one day of monoculture. For control monocultures, MRC-5 cells seeded on transwell inserts were grown in wells without PDAC cells, whereas PADC cells were grown using empty inserts. From this moment on, media were supplemented with the TGF-β and its inhibitors according to the different experimental conditions. Co-cultures and monocultures in transwells/wells were so maintained in incubation for the following 5 days, which represents the time limit that can be reached to avoid cell sufferance into the transwell/well systems, before proceeding with the following analyses.

### 2.4. Cell Proliferation Assays

MRC-5 cells cultured on the transwell in the absence or presence of TGF-β (5 ng/mL), with or without the TGF-β signaling pathway inhibitors of TGF-βR1 or SMAD3, or in the presence of PANC-1 cells with or without the same inhibitors, were collected from the upper side of the insert and counted. The same process was performed with PANC-1 cells cultured for 5 days in the 12-well multiplate, in the absence or presence of TGF-β, or in the presence of MRC-5 cells, with or without the TGF-βR1 or SMAD3 inhibitors.

### 2.5. Western Blots

Western blot was used to detect the expression of α-SMA in activated fibroblasts, E-cadherin and N-cadherin in tumor cells, and phosphorylated SMAD2/3 in both cell lines, at the end of the transwell culture, compared to the monoculture without or with 5 or 10 ng/mL TGF-β.

MRC-5 cells were detached from the inserts with trypsin and, after three washes with cold PBS, collected by centrifugation. They were then lysed using a cell lysis buffer consisting of RIPA and Halt™ Protease Inhibitor Cocktail (78438, Thermo Fisher Scientific, Monza, Italy). For the analysis of phosphorylated SMAD2/3, the phosphatase inhibitors (sodium fluoride and sodium orthovanadate) [32] were also added to the lysis buffer. Then, the lysates were incubated at 4 °C for 30 min on a mechanical wheel for continuous mixing of buffer and sample and, at the end of the incubation, the samples were centrifuged at 4 °C at 13,000× *g* for 15 min and the supernatant collected for the protein dosage using a bicinchoninic acid assay (BCA). The absorbance values were read with an EnSight™ Multimode Plate Reader (PerkinElmer, Milan, Italy).

For the analysis of E-cadherin and N-cadherin, and of phosphorylated SMAD2/3 in PANC-1 cells, the cells were lysed into the well with the same lysis buffers described above, removed from the surface of the well using a cell scraper, and placed in tubes with low surface protein adhesion.

For WB analysis, 12% acrylamide gel was used for anti-α-SMA, and 8% acrylamide gel was used for anti-N-cadherin and E-cadherin. Glyceraldehyde 3P dehydrogenase (GAPDH) with a molecular weight of 36 kDa was used as the housekeeping gene; 8% acrylamide gel was also used for phosphorylated SMAD2/3 detection, and SMAD2/3 was also blotted to provide a reference of the total protein content.

After the transfer, the PVDF membrane was blocked with TBS Tween20 0,1% BSA 5% for 1 h, and then blotted for 2 h with the following primary antibodies in a blocking solution: 0.5 μg/mL anti-α-SMA (rabbit, ab5694, Abcam, Cambridge, UK), anti-N-cadherin (mouse, MA1-91128, Thermo Fisher Scientific, Monza, Italy), 1:1500, anti-E-cadherin (rabbit, 24E10, Cell Signaling, by Euroclone, Milan, Italy), 1:1000, anti-GAPDH (mouse, ab8245, Abcam, Cambridge, UK), 1:5000, anti-P-SMAD2/3 (rabbit, 8828, Cell Signaling, by Euroclone, Milan, Italy), 1:1000, and anti-SMAD2/3 (rabbit, 8685, Cell Signaling, by Euroclone, Milan, Italy), 1:1000. HRP-conjugated anti-rabbit or anti-mouse secondary antibodies (Cell Signaling, by Euroclone, Milan, Italy), 1:2000, were used before the chemiluminescent reaction with ECL star (Euroclone, Milan, Italy), or SuperSignal™ West Femto (Thermo Fisher Scientific, Monza, Italy) and analyzed using Odissey imaging systems (LI-COR Biosciences, Lincoln, NE, USA). Fiji (ImageJ-win64) was used for densitometric analysis of the acquired images.

### 2.6. MTS Assay

The effect on PANC-1 or MIA PaCa-2 tumor cells of exposure to 50 μM gemcitabine (Gem) in the co-culture with MRC-5 cells or in the monoculture with or without TGF-β (5 ng/mL) was evaluated using the MTS viability test. This colorimetric assay involves the use of a tetrazolium salt [3-(4,5-dimethylthiazol-2-yl)-5-(3-carboxymethoxyphenyl)-2-(4-sulfophenyl)-2H-tetrazolium, saline form, MTS] (CellTiter 96^®^ AQueous One Solution Cell Proliferation Assay, G3582, Promega Corporation, Milan, Italy). Untreated PANC-1/MIA PaCa-2 cells were used as a control. After 7 days of growth on multiwells (of which 5 were in a co-culture with MRC-5 or incubated with TGF-β), tumor cells were detached, counted and re-plated in 6-well multiwells (75,000 cells/well) and incubated for 24 h. Next, the cells were treated with 50 µM Gem or less, and then incubated for 48 h. After this time, the MTS test was performed: Amounts of 500 µL of fresh medium and 100 µL of MTS were added to each well. After 2 h and 30 min of incubation, the solution was transferred to 24-well plates which were analyzed with EnSight™ by measuring the absorbance at a wavelength of 490 nm.

### 2.7. Immunofluorescence of Cadherins

The expression of N-cadherin and E-cadherin proteins in the PANC-1 cell monoculture or cells previously grown in the transwell with MRC-5 cells or TGF-β was evaluated by immunofluorescence. After the transwell culture, tumor cells were detached using trypsin and re-plated at 6000 cells/cm^2^ in 35 mm diameter CELLview Cell Culture Dish with Glass Bottom petri dishes (Greiner Bio-One, distributed by Euroclone, Milan, Italy). After 48 h, cells were fixed in 4% paraformaldehyde PBS 1× for 20 min and then treated with 10 µM of Hoechst 33,342 dye (62249, Thermo Fisher Scientific, Monza, Italy) for 10 min, for nuclear staining. They were subsequently incubated for 1 h with the blocking solution (BSA 2% in PBS 1×) and then with the same primary antibodies, anti-N-cadherin (1:250) and anti-E-cadherin (1:1600), used for WB. As a reference for the localization of cadherins, membranes were also labeled using the anti-CD44 antibody (rat, MCA4703, Bio-Rad Laboratories, Milan, Italy), 1:40. Subsequently, the cells were incubated for 1 h with the secondary antibodies (1:400) anti-rat Alexa Fluor 647 (A48265, Thermo Fisher Scientific, Monza, Italy), anti-mouse Alexa Fluor 555 (A27028, Thermo Fisher Scientific, Monza, Italy) or anti-mouse Alexa Fluor 488 (A-11001, Thermo Fisher Scientific, Monza Italy), and anti-rabbit Alexa Fluor 546 (A11035, Thermo Fisher Scientific), to detect CD44, N-cadherin, and E-cadherin, respectively. Both primary and secondary antibodies were diluted in the blocking solution. The cells were then stored in PBS at 4 °C until analysis. A Nikon A1R confocal fluorescence microscope (Nikon Instruments, Melville, NY, USA) was used for image acquisition (single or z-stack acquisition modes), using the following lasers: 405 nm to detect Hoechst, 488 nm or 543 nm to detect N-cadherin, 543 nm to detect E-cadherin, and 633 nm to detect CD44. Image elaboration, z-stack projection, and mean fluorescence intensity quantification were obtained with Fiji (ImageJ-win64).

### 2.8. Wound-Healing and Migration Assays

The migratory activity of PANC-1 cells exposed to MRC-5 fibroblasts in the transwell or to TGF-β (5 ng/mL) was assessed by a wound-healing assay, using culture-insert petri dishes (Culture-Insert 3 Well, Ibidi, by Twin Helix, Milan, Italy). The collected cells were seeded in DMEM at a density of 6 × 10^4^/cm^2^ inside the 3 small chambers of the petri dish containing the silicon insert. After 24 h, the insert was removed. The fresh medium was added and the migration of the cells into the cell-free space was detected at 4, 8, and 24 h by means of the Paula imaging system. The cell-free space was measured over time, starting from the initial distance of 500 μm.

With the same PANC-1 cell samples reported above, migration assays were performed by seeding the cells on the membrane of a 12-well Cell Culture Insert, 8 µm pore size (ThinCert^®^—Greiner Bio-One, by Euroclone, Milan, Italy). The cells (2 × 10^5^) were seeded in 400 µL of medium without FBS into the upper chamber the transwell system, and the same medium was also added into the lower chamber. After 24 h of incubation at 37 °C, the DMEM of the lower chamber was replaced with the same medium with 10% FBS added. After a further 48 h, the upper chamber medium was aspired and the non-migrated cells remaining on the upper surface of the transwell membrane were gently scraped with a cotton swab. The colorimetric method CytoSelect™ Cell Migration Assay (Creative Bioarray, by DBA Italia, Milan, Italy), which includes cell staining, dye extraction, and spectrophotometric quantification, was used to detect the cells that passed into the lower chamber. To this end, the inserts were transferred into wells containing the staining solution and, after 10 min at RT, extensively washed in water and then put into the extraction solution. After another 10 min, 100 µL of the samples was quantified with EnSight™ at a wavelength of 560 nm.

### 2.9. Monospheroid and Heterospheroid Production, and Paula Imaging

Heterospheroid production was obtained by seeding 3000 PANC-1 cells and 3000 MRC-5 cells together with MRC-5 cells in a 96-well nucleosphere plate with a hemispherical bottom (Nunclon™ Sphera™ 96-Well, Thermo Fisher Scientific, Monza, Italy) in either 100 μL or 200 μL, the latter for detecting IL-6 in supernatants by ELISA, of RPMI/DMEM (1:1). After centrifugation of the plate at 1300 rpm for 10 min, the produced spheroids were observed with the Paula microscope. Control monospheroids were produced using MRC-5 or PANC-1 cells with their own culture medium (3000 cells/well). Media were then supplemented with the TGF-β with or without its inhibitors for the monospheroids, or only with the inhibitors for the heterospheroids. Hetereospheroids and monospheroids were kept for up to 7 days and then analyzed with the Paula digital microscope to assess the effect of treatments on their diameter.

### 2.10. Image Analysis of Spheroids

To double-label the heterospheroids, the two cell types, PDAC (PANC-1 or MIA-Paca-2) and MRC-5 cells, were incubated with Vybrant^®^ CFDA SE (λex = 492, λem = 517 nm) and CellTracker™ Deep Red dye (λex = 630, λem = 660 nm) (Invitrogen, Oxford, UK), respectively, before making the heterospheroids. After 7 days of co-culture in the nucleosphere, the produced heterospheroids were fixed in 4% paraformaldehyde for 10 min, washed in PBS 1×, and then transferred to a 96-well multiwell plate with a flat black base (Greiner Bio-One, by Euroclone, Milan, Italy) to analyze their fluorescence signal. PANC-1-based spheroids were analyzed with Thunder Imager 3D Live Cells (Leica, Milan, Italy), whereas the much larger MIA-based spheroids were analyzed using an Operetta CLS fluorescence microscope (Operetta CLS High Content Analysis System—PerkinElmer, Milan, Italy) with a 5× objective. All obtained images represent a single central section and were processed with Fiji (ImageJ-win64).

For the invasiveness assay, PANC-1 cells labeled with Vybrant^®^ CFDA SE were used to produce monospheroids with or without 5 ng/mL TGF-β or heterospheroids with unlabeled MRC-5. After 4 days from seeding in the nucleosphere, 100 µL of medium was substituted with the same amount of a murine Engelbreth–Holm–Swarm sarcoma matrix (Cultrex^®^, 3-D Culture Matrix Reduced Growth Factor BME, R&D SYSTEM, by Bio-Techne, Milan, Italy). The matrix, thawed on ice, was added to the spheroids into the nucleosphere plate, and, after 1 h, 100 µL of fresh medium was added. After the following 3 days, all mono- and heterospheroids were analyzed with Operetta using brightfield and AF488 channels and the images, representing a single inner z-stack, were analyzed with Operetta Harmony™ software (version no. 5.2.3). With the function “find region”, we determined the region of analysis based on the AF488 channel, corresponding to the main spheroid body and other minor objects related to proliferating cells invading the surrounding matrix. Using the function of the “common threshold” method, we set the threshold of separation of the objects to 0.35–0.5, and the minimum detected area to 20 µm. Using the option “calculate morphology properties”, the following morphology parameters related to the main spheroid body were determined: spheroid area (µm^2^), roundness, width/length ratio, and number of all small fluorescent objects corresponding to the spreading cells (n. cells in the surrounding area).

The growth of the heterospheroids was confirmed by live-cell imaging using Incucyte SX5 (Sartorius, Göttingen, Germany). Brightfield images were acquired every 8 h at 4× magnification, and the spheroid area was measured using the built-in software. This analysis revealed that the heteroshperoids grow over time.

### 2.11. Cytotoxicity on Spheroids: LDH Assay

The cytotoxicity of gemcitabine on the mono- and heterospheroids was assessed by measuring, with the CyQUANT LDH Cytotoxicity Assay kit (C20300, Invitrogen, Carlsbad, CA, USA), the enzyme lactate dehydrogenase (LDH) released from the cell cytosol due to the damage to the cell membrane.

After 48 h of incubation with Gem in 200 uL of medium in the nucleosphere plate, 150 μL was removed from each well and the spheroid with the remaining supernatant was treated with 10 μL of a lysis buffer provided with the kit, and then incubated for 45 min at 37 °C to promote lysis. Subsequently, 50 μL of both the lysates and the supernatants were transferred to a 96-well plate and 50 μL of the reaction mix was added to each sample and incubated for 30 min at RT. Consequently, the reaction was stopped and absorbance was measured at wavelengths of 490 and 680 nm. The difference between the signals at 490 nm and 680 nm is proportional to the amount of LDH present in the sample. Data were then processed and expressed as the percentage of death in cells (C) exposed to gemcitabine with respect to untreated cells, according to the following equation:

% drug-induced death C = [1 − (Lysate LDH treated/Lysate LDH control)] × 100

This equation allows us to eliminate interferences due to the spontaneous release of LDH in untreated populations which cause the underestimation of the true signal resulting from chemotherapy-induced death in the treated population [33]. The quantity of LDH in the lysates was subtracted by the signal of the supernatant, both previously deprived of the intrinsic signal of the medium.

### 2.12. Quantification of TGF-β and IL-6 by ELISA

The TGF-β released into the culture medium was quantified by ELISA. The conditioned medium taken from the lower chamber of the transwells or from spheroids was collected and centrifuged at 12,000 rpm for 10 min at 4 °C. The supernatants were maintained at −20 °C to be then analyzed with a TGF-β1 Human/Mouse Uncoated ELISA kit (Thermo Fisher Scientific, Milan, Italy). The same procedure was also applied to the culture medium of the transwell (upper and lower chambers) for the measurement of interleukin-6 (IL-6) secreted by fibroblasts in the mono- or co-culture using the IL-6 Human Uncoated ELISA Kit (Thermo Fisher Scientific, Milan, Italy). The 96-well plate was read with EnSight™ at wavelengths of 450 nm and 570 nm.

### 2.13. Statistical Analysis

All statistical analyses were conducted by one-way ANOVA with multiple comparison using Tukey’s multiple comparisons test (GraphPad Prism 9 software). The normality of the distribution was checked with the post hoc Shapiro–Wilk test before the ANOVA was performed. Values of *p* < 0.05 were considered statistically significant. All groups were compared with each other. Only statistical differences are reported.

## 3. Results and Discussion

### 3.1. PDAC Cells Promote the Activation of Fibroblasts via TGF-β Secretion

**Figure 1:**
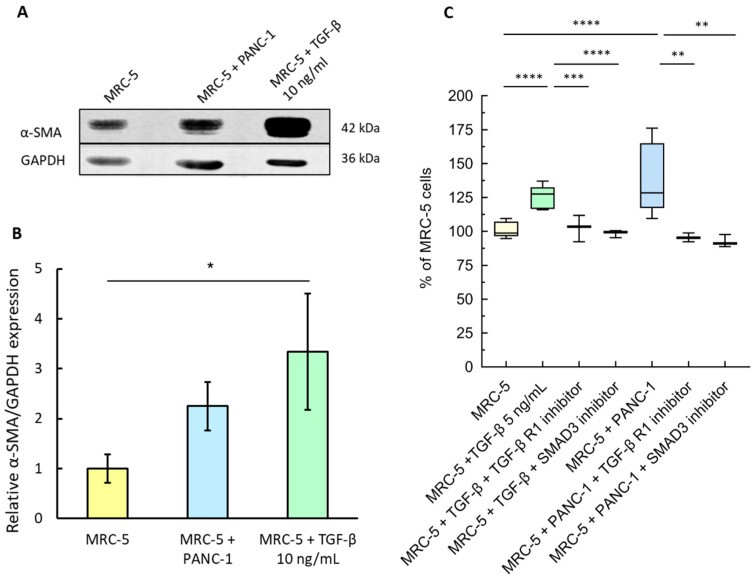
TGF-β released by tumor cells promotes fibroblasts’ activation. (**A**) Expression of α-SMA in MRC-5 fibroblasts, cultured on the upper side of the transwell, at the end of 5 days exposure to PANC-1 cells, or TGF-β (10 ng/mL); representative cropped images of the Western blot of α-SMA and GAPDH (housekeeping protein) derived from the original blots in Figure S1. (**B**) The intensity of the SMA signal was quantified and normalized to that of GAPDH from the same lane (α-SMA expression in MRC-5 cells in the monoculture was set to 1. Mean values ± SE (n = 3 biological replicates) were compared by one-way ANOVA; * *p* < 0.05. (**C**) MRC-5 fibroblasts at day 5 of the monoculture or treated with TGF-β (5 ng/mL), with or without 10 μM TGF-β receptor inhibitor (SB431542) or SMAD3 inhibitor (SIS3), or day 5 of the co-culture with PANC-1 cells, in the absence or presence of the same inhibitors. The number of MRC-5 cells in the monoculture was set as a reference at 100%. Data, represented as box-and-whisker plots, were compared by one-way ANOVA; **** *p* < 0.0005, *** *p* < 0.001, ** *p* < 0.01 (n = 3–13).

The activation of MRC-5 fibroblasts exposed to PANC-1 tumor cells was initially determined by assessing the protein levels of alpha-smooth muscle actin (α-SMA), a well-established marker of CAFs, particularly of myCAFs. The expression of α-SMA was evaluated in MRC-5 fibroblasts cultured on the upper side of transwell units for 7 days, either as a monoculture with or without TGF-β or as a co-culture with PANC-1 cells. TGF-β was used as a positive control, as it is a known soluble mediator responsible for the reprogramming of normal fibroblasts into CAFs. α-SMA was expressed in the control MRC5 monocultures and its levels increased upon treatment with TGF-β. More importantly, α-SMA expression was more than doubled compared to the control when MRC-5 cells were grown together with PANC-1 cells (Figure 1A,B). This result shows that the PANC-1 pancreatic adenocarcinoma cells can activate MRC-5 fibroblasts, leading to the upregulation of the CAF marker protein α-SMA, just like TGF-β does. In PDAC myCAFs, the high expression of α-SMA is associated with a low expression of IL-6 [34]. Instead, IL-6 is highly expressed in iCAFs, which have low αSMA levels [1,26], and are induced by IL-1 through the JAK/STAT pathway [35]. IL-6 was not upregulated in MRC-5 cells co-cultured with PANC-1 cells (Figure S2), which is in line with the acquisition of a myCAF-like phenotype. Importantly, we have previously demonstrated that the release of soluble IL-6 by αSMA-negative iCAFs was activated by triple-negative breast cancer (TNBC) cells co-cultured in the same transwell model in comparison to control fibroblasts [31].

Activated fibroblasts show an increased proliferation compared to normal fibroblasts, and the ability of TGF-β to promote fibroblasts’ proliferation has already been demonstrated in different settings [9,31]. Thus, we compared the MRC-5 cells co-cultured with the PANC-1 cells with the MRC-5 monocultures using the transwell model, which allows only paracrine signaling. We found that the number of MRC-5 cells co-cultured with the PANC-1 cells was significantly higher than that of the monoculture (Figure 1C), in agreement with the effects of TGF-β, which was utilized as a positive control. Furthermore, this increase was significantly inhibited upon exposure to the TGF-βR1 or the SMAD3 inhibitor, in both monocultures and co-cultures, suggesting the major role of this growth factor in fibroblast activation (Figure 1C). These data also support that activated fibroblasts have a myCAF phenotype [14], which is characterized by the activation of the TGF-β/SMAD2/3 pathway [34]. SMAD2/3 phosphorylation was slightly enhanced in the MRC-5 cells co-cultured with the PANC-1 cells with respect to the MRC-5 monocultures (Figure S3), corroborating the involvement of the TGF-β/SMAD2/3 pathway.

MRC-5 hyperproliferation was also observed when these cells were co-cultured with another human PDAC cell line, MIA Paca-2 (Figure S4), confirming that this TGF-β-mediated mechanism is conserved among different tumor cells.

### 3.2. TGF-β Secretion by Activated Fibroblasts Stimulates PDAC Cells’ Growth and Chemoresistance

Previous studies have described that PDAC progression, invasion, and/or chemoresistance are supported by CAF activity, and that the TGF-β released into the microenvironment by these cells plays a major role in these responses [22]. Using the PDAC-MRC-5 cell co-culture system, we aimed to verify if there was a TGF-β signaling loop between these two cell types.

**Figure 2:**
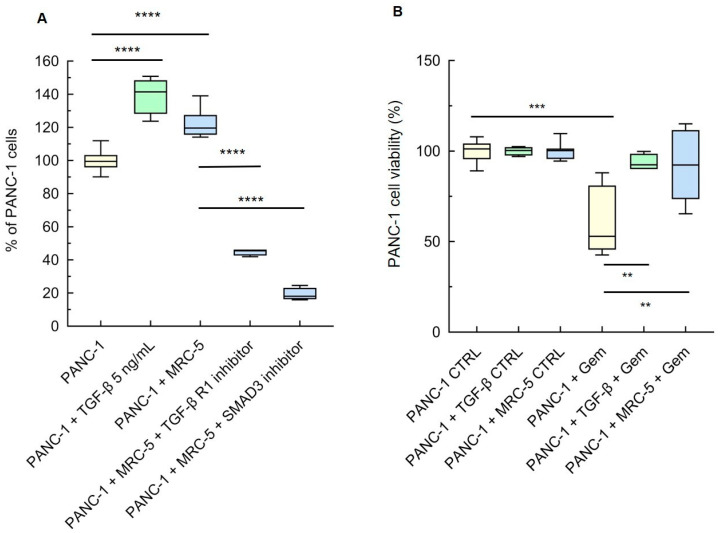
(**A**) MRC-5 cells promote tumor-cell proliferation in transwell co-cultures. PANC-1 cells in the lower chamber of the transwell units were counted after 5 days of co-culture with MRC-5 cells or exposure to TGF-β (5 ng/mL), with or without 10 μM TGF-β receptor inhibitor (SB431542) or SMAD3 inhibitor (SIS3). The number of PANC-1 cells in the monoculture was set as a reference at 100%. (**B**) MRC-5 cells promote tumor-cell chemoresistance to gemcitabine (Gem). The viability of PANC-1 cultured for 5 days with or without MRC-5 fibroblasts in the transwell, or with or without TGF-β (5 ng/mL), and then treated with 50 µM Gem for 48 h, was assessed by MTT. The viability of the untreated monoculture was set to 100%. Data, represented as box-and-whisker plots, were compared by one-way ANOVA. ** *p* < 0.01, *** *p* < 0.001, **** *p* < 0.0005 ((**A**): n = 4–10; (**B**): n = 4).

We compared the proliferation of PANC-1 cells that were co-cultured in the transwell model with MRC-5 fibroblasts, or treated with TGF-β (5 ng/mL) as a positive control, to that of the untreated monocultures. The number of tumor cells grown with TGF-β was significantly increased with respect to the untreated monocultures, as expected (Figure 2A) [18]. The same occurred when the PANC-1 cells were grown with MRC-5 fibroblasts (Figure 2A). An even more prominent increase was observed in the MIA Paca-2 cells exposed to MRC-5 fibroblasts (Figure S5). This is at variance with the inhibitory effect exerted by the MRC-5 cells’ conditioned medium on the proliferation of PDAC cells. The discrepancies could stem from the different end point (5 vs. 14 days) [36]. To corroborate that the TGF-β released by activated MRC-5 into the medium is responsible for tumor-cell hyperproliferation, PANC-1 cells were cultured with MRC-5 cells with the TGF-βR1 or SMAD3 inhibitor, added in the lower chamber; both inhibitors markedly reduced the number of cancer cells (Figure 2A). As for the MRC-5 cells, we found that the phosphorylated levels of SMAD2/3 were increased in the PANC-1 co-cultured with fibroblasts compared with those in the PANC-1 monocultures (Figure S3). Of note, the co-culturing boosted the activity of the TGF-β/SMAD2/3 pathway more in the PANC-1 than the MRC-5 cells.

In PDAC, as in other cancer models, tumor progression and metastasis are often associated with CAF-induced chemoresistance [8,13], and TGF-β has been identified as one of the cytokines involved, acting directly through the TGF-β/SMAD pathway or indirectly through pro-survival pathways [21]. The sensitivity of PANC-1 cells to the chemoterapeutic gemcitabine (Gem) was determined in the transwell co-culture system and compared to that of the monocultures. TGF-β added into the lower chamber was used as a positive control. These experiments showed that the viability of the PANC-1 cells treated with 50 µM Gem for 48 h was halved compared to the control culture (Figure 2B). Strikingly, the PANC-1 cells co-cultured with the fibroblasts became fully resistant to Gem (Figure 2B).

Although IL-6 is known to enhance the invasiveness and chemoresistance of different cancer types [37,38,39,40], and to be upregulated in CAFS in response to the signals released by desmoplastic cancer cells [31], we found that IL-6 released in the lower chamber (25.13 ± 0.705, mean ± SE, n = 4) was 24-fold less abundant than TGF-β (Table S1). This and the above experiments collectively suggest that the TGF-β released in the lower chamber of the co-culture system is a key factor activating cancer cells. Yet we cannot exclude that additional paracrine factors secreted by the activated fibroblasts may contribute to the observed phenotypes.

### 3.3. The Effect of Activated Fibroblasts on the EMT in PDAC Cells

CAFs often induce cancer cells to undergo EMT, which is a result of TGF-β signaling in PDAC cells and enhances their migratory abilities [17,18].

**Figure 3:**
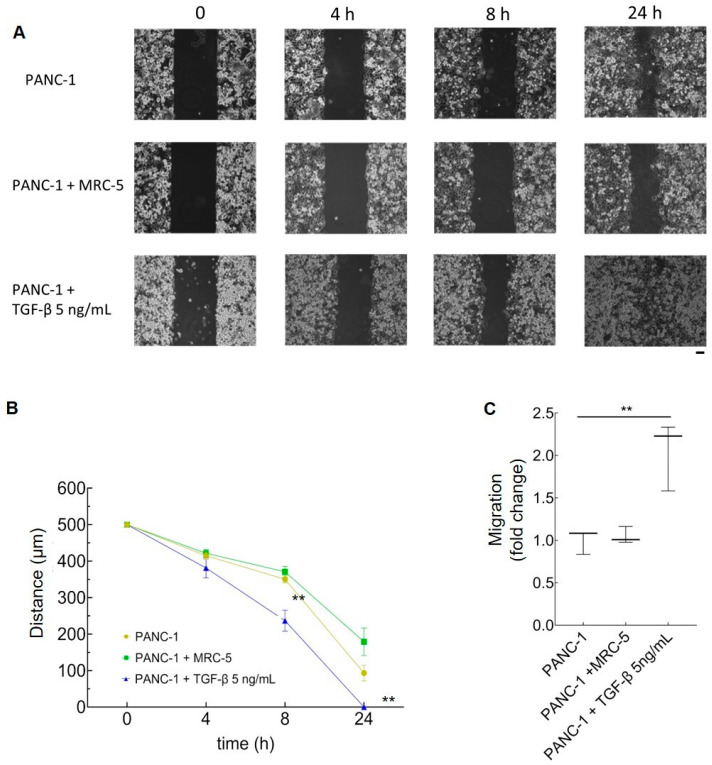
Migration of PANC-1 cells upon culture with activated fibroblasts or TGF-β. (**A**) Wound-healing assays performed with PANC-1 cells at the end of 5 days of culture in the presence of 5 ng/mL TGF-β, or co-cultured with MRC-5 cells, to detect their migration at 4, 8, and 24 h; starting cell-free space: 500 μm. Bar: 100 µm. (**B**) Migration-free space (μm) over time (0–24 h). Points represent mean values ± SE (n = 3) and were compared by one-way ANOVA (** *p* < 0.01 vs. control PANC-1). (**C**) Migration ability of PANC-1 cells induced by 5 ng/mL TGF-β or co-cultured with MRC-5 cells in transwell units. The migration of PANC-1 cells alone was taken as a reference (1) to normalize the data. Mean ± SE (n = 3) are displayed. ** *p* < 0.01 (one-way ANOVA).

To assess the migration of PANC-1 cells cultured in the transwell in the absence or presence of MRC-5, wound-healing assays were performed and the migration of the PANC-1 cells into the wounded area was monitored over time (Figure 3A). TGF-β promoted cell migration and wound closure with respect to the control PANC-1 cells, as expected [17] (Figure 3B). Surprisingly, the MRC-5 fibroblasts had no such effect on the PANC-1 cells and seemingly decreased the migration of tumor cells (Figure 3A,B). These data are in line with previous studies showing that MRC-5 cells’ conditioned medium impairs the migratory and invasive abilities of cultured pancreatic tumor cells [36].

Migration assays in which PANC-1 cells were grown with or without MRC-5 or TGF-β as a positive control showed that only TGF-β stimulated the motility of PANC-1 cells (Figure 3C). This agrees with the results of the wound-healing assays and suggests that the TGF-β released by MRC-5 cells is able to stimulate the proliferation of the PANC-1 cells, but not the migration ability of these cells.

The expression of E-cadherin and N-cadherin, established EMT markers, in PANC-1 cells was analyzed in the monoculture and upon MRC-5 exposure, in comparison to tumor cells exposed to TGF-β. We observed that the expression of E-cadherin in PANC-1 cells was highly responsive to TGF-β, with an evident downregulation in the cells co-cultured with MRC-5 for 5 days (Figure S6A). Conversely, that of N-cadherin did not show any increase in either the co-culture or the monoculture upon TGF-β treatment (Figure S6B).

However, the subcellular localization of both cadherins was affected by the culturing conditions. Confocal microscopy showed that PANC-1 cells co-cultured with fibroblasts or treated with TGF-β had lower E-cadherin levels compared to the monoculture (Figure S7A). Concerning N-cadherin, a certain positivity was observed on the plasma membrane of some of the PANC-1 cells co-cultured with MRC-5 as opposed to the robust fluorescent signal found in most of the cells treated with TGF-β (Figure S7B). Compressed z-stack projections further revealed that E-cadherin no longer localizes on the plasma membrane of PANC-1 cells treated with TGF-β or co-cultured with MRC-5c fibroblasts, and that its staining intensity significantly decreases with respect to the PANC-1 monoculture. The intensity and localization of the N-cadherin showed the opposite trend instead (Figure S7C). Although the TGF-β released by the MRC-5 cells does not induce a full EMT in PANC-1 cells, it is enough to change the cell-surface levels of both E-cadherin and N-cadherin. As previously reported [36], factors secreted by the MRC-5 cells could impair the migration of pancreatic cancer cells in which the EMT program is activated. On the other hand, the significant activation of migration in the tumor cells exposed to high doses of TGF-β, such as the one that we used, confirms the role of this factor in the EMT process.

### 3.4. SMAD3-Mediated But Not TGF-β-Mediated Signaling Impacts the Size of MRC-5/PANC-1 Heterospheroids

**Figure 4:**
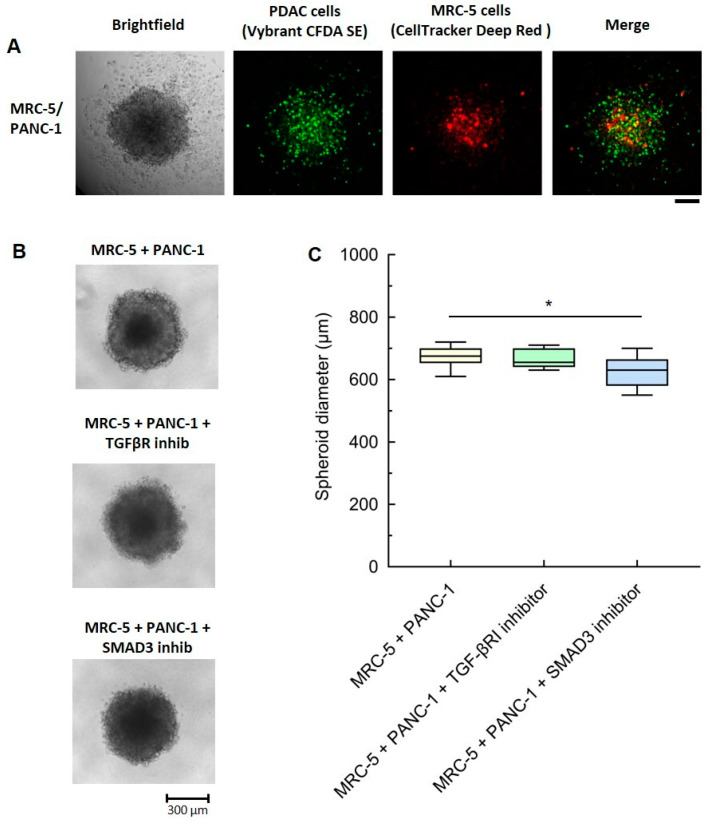
Role of TGF-β in PANC-1/MRC-5 heterospheroid proliferation. (**A**) Representative images of heterospheroids consisting of PANC-1 and MRC-5 cells pre-labeled with Vybrant^®^ CFDA SE and CellTracker™ Deep Red dye. Scale bars: 200 µm. (**B**) Images of MRC-5/PANC-1 heterospheroids treated with 10 μM TGF-βR1 inhibitor (SB431542) or SMAD3 inhibitor (SIS3) for 7 days. (**C**) The size of the spheroids was determined using the acquired images (average of 4 different diameters for each condition). Data, represented as box-and-whisker plots, were compared by one-way ANOVA. * *p* < 0.05 (n = 10–12).

Heterospheroids consisting of MRC-5 and PANC-1 were developed to allow for both paracrine and cell-contact-based signaling. To determine the distribution of the different cell types into the heterotypic 3D tumor spheroid, the two cell types were labeled with different fluorescent dyes (Figure 4A). Seven-day-old heterospheroids displayed a rather even distribution of MRC-5 and PANC-1 cells within the spheroids, with the former being slightly more concentrated in the core with respect to the latter (Figure 4A).

By measuring the dimension of the spheroids (average diameter), we discovered that SMAD3 and the TGF-βR1 inhibitors had a minor and no impact, respectively (Figure 4B,C). An even more marked effect was observed in MIA cell-based heterospheroids, whose dimension was much larger, and where the two cell types were distributed more uniformly (Figure S9). Of note, MRC-5 and PANC-1 monospheroids showed an increased diameter upon exposure to TGF-β, but neither inhibitor produced an effect (Figure S8).

The smaller size of the heterospheroids treated with the SMAD3 inhibitor is thus the result of the lower proliferation of the cancer cells and fibroblasts, in agreement with the response observed in the transwell system (Figure 1C and Figure 2A). The lack of effect observed in the heterospheroids exposed to the TGF-βR1 inhibitor suggests, however, that it is not so much the TGF-β signaling pathway that affects cell proliferation into the spheroid, but rather other signaling pathways convergent on SMAD3. It is worth noting that the amounts of TGF-β released by the heterotypic spheroids made by PANC-1 and MRC-5 and by the monotypic PANC-1 spheroid were similar (about 2 ng/mL), and lower than that used as a positive control. The amount of TGF-β released by MIA-Paca-2 was even lower (0.7 and 0.9 ng/mL, respectively) (Table S1). The TGF-β released by the monotypic MRC-5 spheroid was 1.4 ± 0.6 ng/mL (mean ± SE, n = 4), suggesting that both MRC-5 and PDAC cells may be a source of TGF-β in the heterospheroids.

### 3.5. Activation of TGF-β Pathways in MRC-5/PANC-1 Heterospheroids Supports Tumor Chemoresistance and Invasiveness

**Figure 5:**
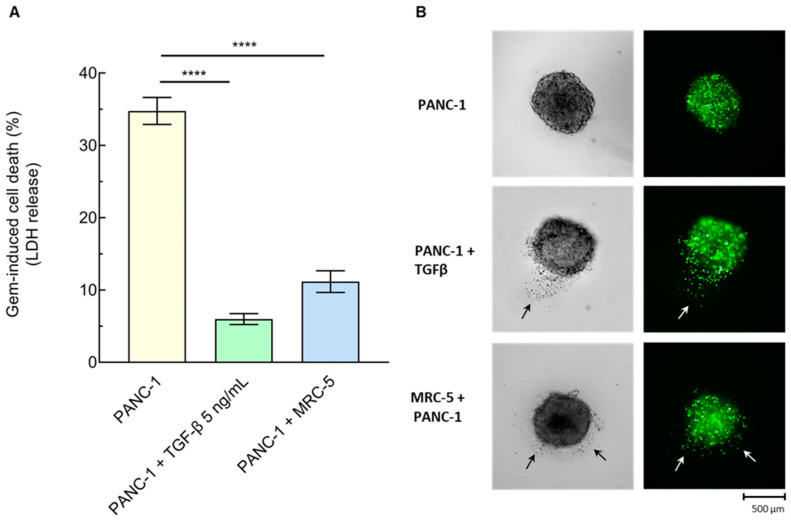
Chemoresistance and invasiveness of PANC-1 cells in heterospheroids, and role of TGF-β. (**A**) Cell death of 7-day-old PANC-1 monospheroids, pre-incubated with or without TGF-β (5 ng/mL), or PANC-1/MRC-5 heterospheroids after 48 h incubation with 50 μM gemcitabine (Gem), determined as LDH production (% variation toward the respective untreated ones). Means ± SE (n = 3–7), **** *p* < 0.0005 vs. PANC-1 monospheroids (one-way ANOVA). (**B**) Invasiveness of PANC-1 cells labeled with Vybrant^®^ CFDA SE from 7-day-old PANC-1 monospheroids, pre-incubated with or without TGF-β, or PANC-1/MRC-5 heterospheroids into the surrounding semi-solid matrix. Spheroids were observed with an Operetta CLS Imaging System (5×), and brightfield and fluorescence images represent a single inner z-stack. Arrows indicate PANC-1 cells spreading from the spheroid to the surrounding area.

Based on the above results, we sought to reassess PDAC chemoresistance and invasiveness in 3D culture settings. Firstly, we tested whether the MRC-5 fibroblasts enhance the PDAC chemoresistance in the 3D heterospheroid model. To this end, we assessed the sensitivity of PANC-1 monospheroids pre-incubated or not with TGF-β and that of PANC-/MRC-5 heterospheroids to gemcitabine using lactate dehydrogenase as a readout of cell cytotoxicity. We documented a remarkable increase in the release of LDH in PANC-1 monospheroids exposed to this chemotherapeutic agent compared to the untreated control, used as a reference (Figure 5), in line with our previous data. More importantly, LDH release was greatly reduced in both the TGF-β-treated monospheroids and the PANC-1/MRC-5 heterospheroids (Figure 5A).

Secondly, we evaluated the invasiveness of PANC-1 monospheroids or PANC1/MRC-5 heterospheroids into the murine Engelbreth–Holm–Swarm sarcoma matrix. This is a process that is often associated with EMT and the ability to remodel the extracellular matrix [41]. Heterospheroids and monospheroids were treated or not with TGF-β for 4 days and then embedded. Three days later, fluorescently labeled PANC-1 cells were capable of invading the surrounding matrix when monospheroids were incubated with TGF-β but not when they were left untreated (Figure 5B). Quantitative image analysis (see Figure S10 for an example) revealed a significant increase in the number of fluorescent spots (PANC-1 cells) in the area surrounding the heterotypic spheroid and the monospheroid exposed to TGF-β compared to the untreated controls, whereas the area, roundness, and width/length ratio of the spheroids showed no significant differences (Table 1).

It is possible that a higher TGF-β concentration within the spheroids as opposed to the one measured in the medium (Table S1) and/or the direct cell–cell contacts account for the different cellular responses given in the transwell vs. spheroid models. In particular, the physical interactions that are possible only in the 3D model have previously been shown to regulate EMT [10,17,37].

## 4. Conclusions

Here we shed light on the complex interplay between the tumor cells of pancreatic adenocarcinoma and their associated fibroblasts, capitalizing on advanced in vitro cellular models to study the impact of indirect (co-culture in transwell) and direct (heterotypic spheroid) cell communication. By means of such models, we characterized the role of TGF-β, which is secreted by both tumor cells and activated fibroblasts. Previous studies have either focused (also in PDAC) on how TGF-β impacts fibroblasts’ activation into CAFs or on the effect on PDAC cells. Conversely, these pathologically relevant yet simple cellular models have allowed us to study the TGF-β signaling loop between fibroblasts and tumor cells in full.

The results obtained from the co-cultures of PDAC cells and MRC-5 human fibroblasts on the transwell confirmed the activation of the fibroblasts and the acquisition of a myCAF-like phenotype, with increased proliferation being dependent on the activation of the TGF-β/SMAD signaling axis. In turn, fibroblast activation made PDAC cells more proliferative and chemoresistant. Indirect communications between the two cell types were insufficient to induce a full EMT in cancer cells or to increase their migratory activity. The use of the embedded 3D spheroid model turned out to be enlightening, because it allowed us to find that tumor-cell motility is increased in heterospheroids. This may be due to the combination of cell-to-cell contacts and paracrine signals. The role of the TGF-β released by MRC-5 on the proliferation of PANC-1 is evident in the transwells. However, the proliferative index of heterospheroids is derived from an indirect measurement (i.e., the diameter of the heterospheroids), and this might obscure the effects induced by medium changes. In line with this, only the SMAD3 and not the TGF-βR1 inhibitor showed a significant, yet mild effect. In any case, these results suggest that other signaling pathways that are convergent on SMAD3, and not only the TGF-β pathway, determine cell proliferation in the spheroid. Conversely, the impact of MRC-5 on PANC-1 chemoresistance appears to be independent of the co-culture model.

From these studies, it seems evident that the complementary use of both the transwell and the spheroid in vitro models provides more relevant information than that gathered from either model alone. In the first case, the transwell makes it possible to analyze each cell type separately, using a simple, flexible, and accessible system. In the second case, the 3D spheroid system not only more closely mimics structures observed in vivo but also allows direct tumor cell–CAF contacts.

So, in the present study, we confirmed the importance of the interactions between tumor cells and stromal fibroblasts in determining the aggressive characteristics of PDAC. Soluble TGF-β certainly has a primary role in the communication process between tumor cells and fibroblasts, albeit in combination with other paracrine factors and other types of interactions, such as the direct contact between tumor and TME cells. TGF-β and its signaling and biosynthetic pathways can represent valid targets for therapies aimed at limiting PDAC progression, metastasis, and chemoresistance.

## Figures and Tables

**Table 1:** Invasiveness and morphometric analysis of mono- and heterospheroids imaged with Operetta CLS, as obtained using Harmony software 5.2.3. Means ± SE. Data were compared by one-way ANOVA. *** *p* < 0.001, **** *p* < 0.0005 vs. PANC-1 monospheroids.

	Spheroid Area (µm^2^)	Roundness	Width/Length Ratio	n. Cells in Surrounding Area
PANC-1	371,736 ± 24,242 (7)	0.709 ± 0.012 (7)	0.689 ± 0.012 (7)	7.14 ± 2.37 (7)
PANC-1 + TGF-β	322,285 ± 4138 (3)	0.648 ± 0.032 (3)	0.707 ± 0.032 (3)	66.67 **** ± 4.86 (3)
PANC-1/MRC-5	351,308 ± 12,843 (4)	0.703 ± 0.040 (4)	0.770 ± 0.033 (4)	41.25 *** ± 7.69 (4)

## Data Availability

The original contributions presented in the study are included in the article/Supplementary Material, further inquiries can be directed to the corresponding authors.

## Supplementary Materials

The following supporting information can be downloaded at: https://www.mdpi.com/article/10.3390/cancers16213705/s1, Figure S1: Western blot of α-SMA expression in MRC-5 fibroblasts, Figure S2: IL-6 released by MRC-5 in monoculture versus co-culture in transwell, Figure S3: SMAD2/3 phosphorylation in MRC-5 fibroblasts and PANC-1 cells co-cultured in transwell, Figure S4: TGF-β released by MIA Paca-2 cells promotes MRC-5 proliferation in transwell, Figure S5: Activated MRC-5 cells promote tumor-cell proliferation in transwell, S6: Western blot detection of E-cadherin and N-cadherin in PANC-1 tumor cells, Figure S7: Immunofluorescence for E-cadherin and N-cadherin in PANC-1 tumor cells, Figure S8: Effect of TGF-β treatment on the dimensions of PANC-1 and MRC-5 monospheroids, Figure S9: Role of TGF-β in MIA PaCa-2/MRC-5 heterospheroid proliferation, Figure S10: Elaboration with Harmony™ software of PANC-1 monospheroid Operetta CLS images; Table S1: TGF-β released into the outer medium by PDAC cells co-cultured with MRC-5 in transwell or spheroids.
