# Supplementary material for: TGF-β Signaling Loop in Pancreatic Ductal Adenocarcinoma Activates Fibroblasts and Increases Tumor Cell Aggressiveness
Source: Cancers (Basel). 2024 Nov 1;16(21):3705. doi: 10.3390/cancers16213705 (PMC11545076; doi:10.3390/cancers16213705)
Supplement: Supplementary file 1 [file cancers-16-03705-s001.zip › cancers-3209184-supplementary.pdf]

cancers

TGF-β Signaling Loop in Pancreatic Ductal Adenocarcinoma Activates Fibroblasts and Increases Tumor Cell Aggressiveness

Results

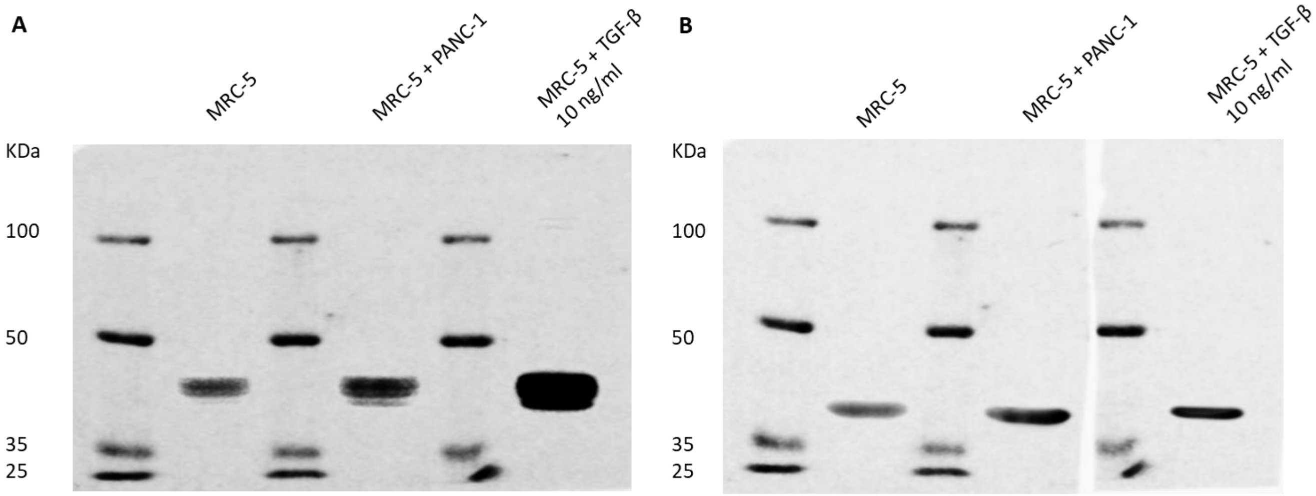

Figure S1. Western blot of α-SMA (A) expression in MRC-5 fibroblasts, cultured on the upper side of transwell, at the end of 5 days exposure to PANC-1 cells, or TGF-β (10 ng/mL), respect to monoculture condition. GAPDH from the same lane (B) has been detected as housekeeping protein. Molecular weights markers are visible on the left of each protein lane.

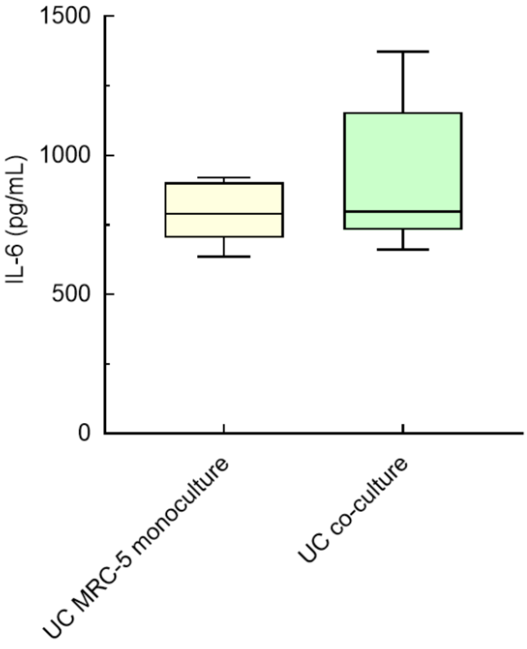

Figure S2. IL-6 (pg/mL) released in the upper chamber of transwell system by MRC-5 in monoculture or co-culture with PANC-1 cells. Data are represented as box-and-whisker plots (monoculture, n = 9; co-culture, n = 11).

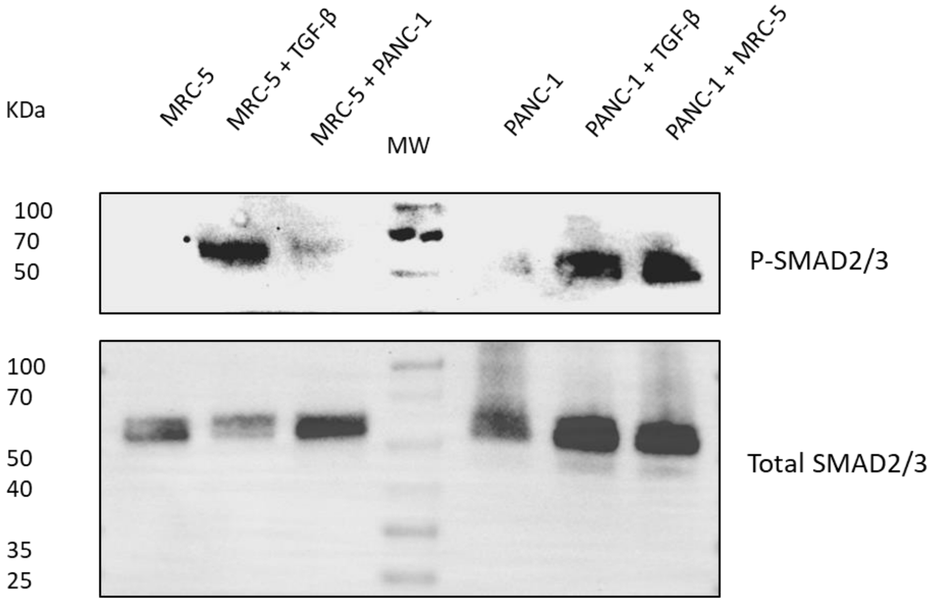

Figure S3. SMAD2/3 phosphorylation in MRC-5 fibroblasts and PANC-1 cells co-cultured in transwell units (upper and lower compartments, respectively) at 5 days, or in monocultures treated or not with TGF-β (5 ng/mL). Total SMAD2/3 served as reference. Molecular weight (MW) markers are visible on the left of each protein lane.

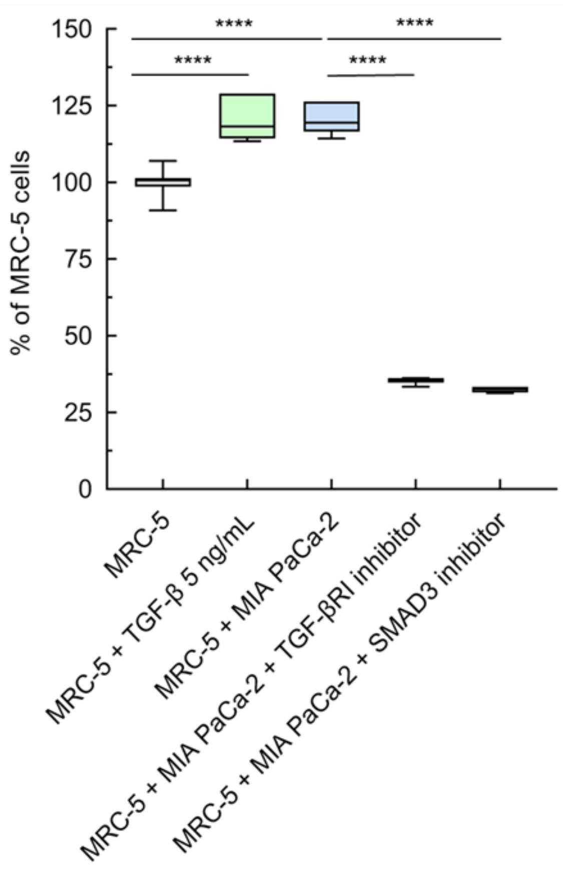

Figure S4. TGF-β released by MIA Paca-2 cells promotes MRC-5 proliferation in transwell. MRC-5 fibroblasts were counted on the upper side of transwell membrane at day 5 of culture, upon exposure to TGF-β (5 ng/mL), or to MIA Paca-2 cells in the absence or presence of 10 μM TGF-β receptor

inhibitor (SB431542) or SMAD3 inhibitor (SIS3) in the upper chamber. The number of MRC-5 cells in monoculture was set as reference at 100%. Data, represented as box-and-whisker plots, are compared by One-way ANOVA **** $p < 0.0005$ ($n = 3–10$).

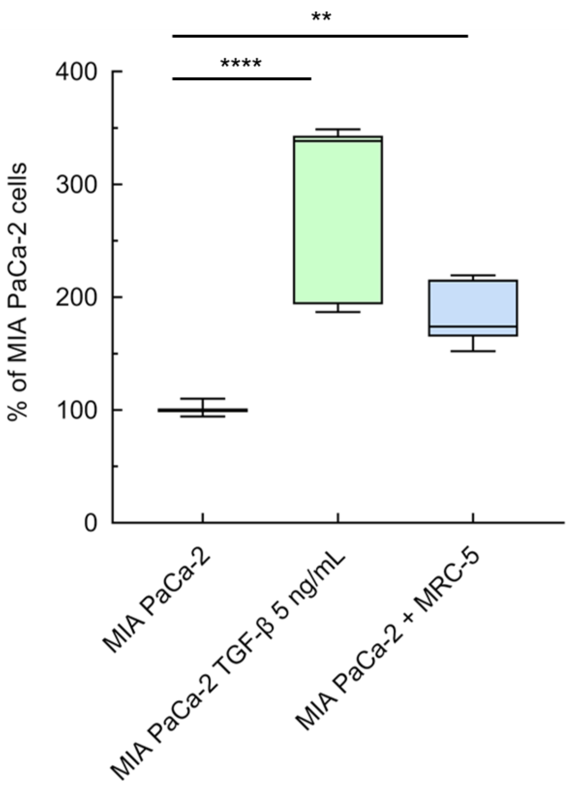

Figure S5. Activated MRC-5 cells promote tumor cells proliferation in transwell. MIA Paca-2 cells counted on the lower chamber of transwell after 5 days of co-culture with MRC-5 or exposure to TGF-β (5 ng/mL). The number of PANC-1 in monoculture was set as reference at 100%. ** $p < 0.01$, **** $p < 0.0005$ (One-way ANOVA, $n = 6-10$).

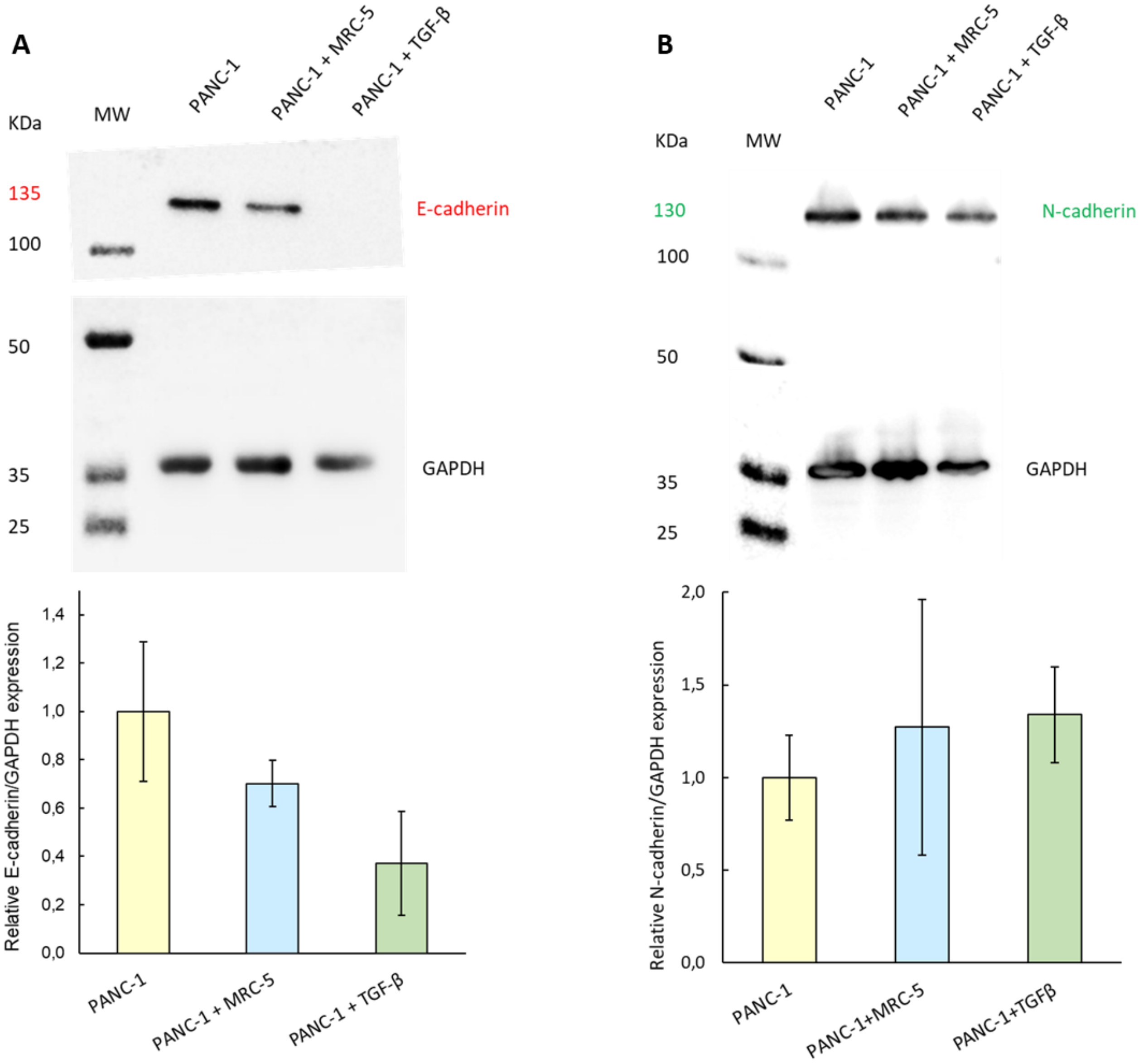

Figure S6. Western blot detection of E-cadherin (A) and N-cadherin (B) in PANC-1 tumor cells. Cadherins' expression was detected after 5 days of culture in the absence or presence of 5 ng/ml TGF-β (positive control), or of co-culture with MRC-5 cells (in transwell). The intensity of the E-cadherin and N-cadherin signals was quantified and normalized to that of GAPDH from the same lane (cadherin expression in PANC-1 cells in monoculture was set to 1). Values are expressed as mean ± SE of 3 (E-cadherin) or 4 (N-cadherin) different biological replicates. MW: molecular weights.

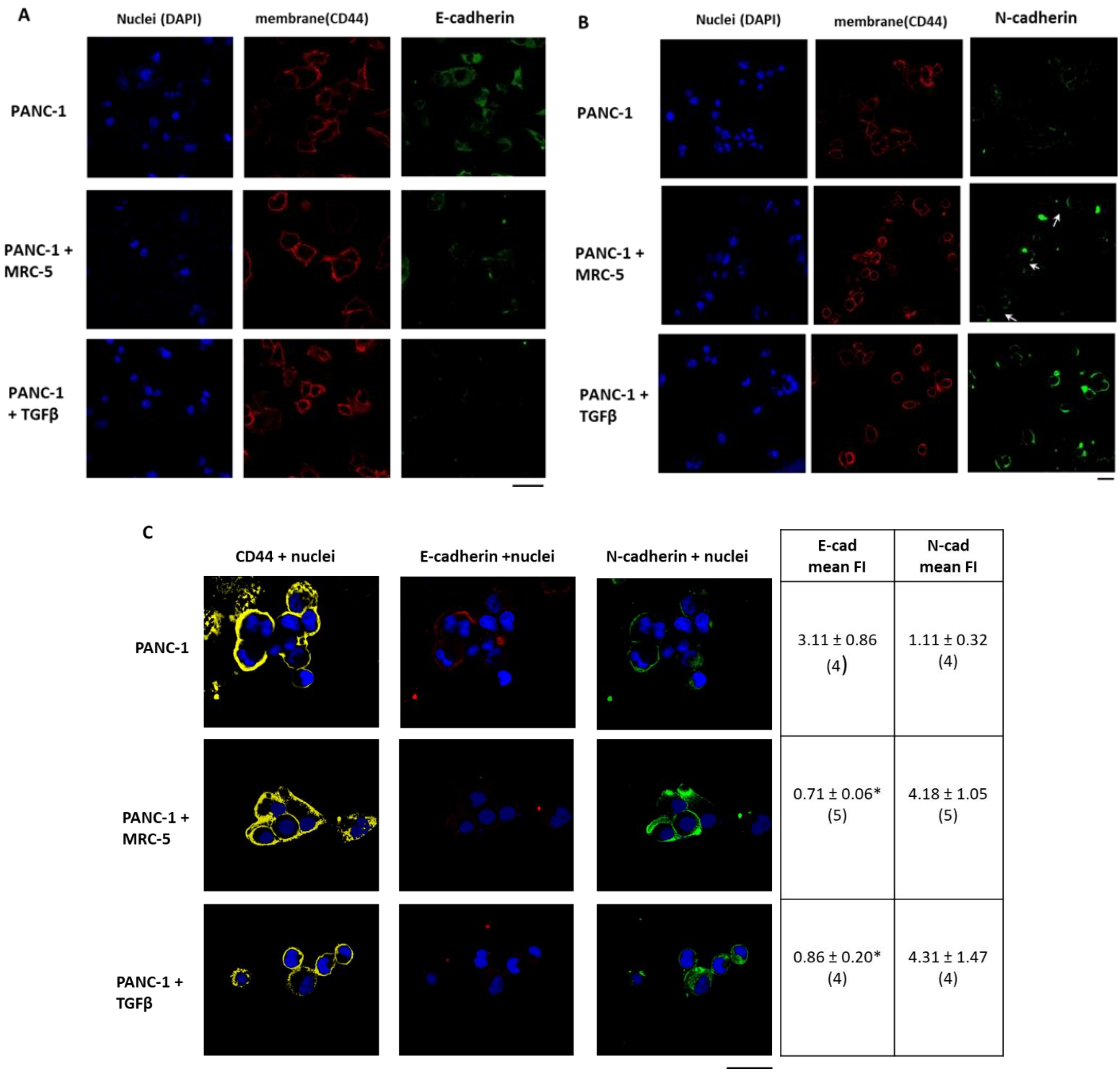

Figure S7. Immunofluorescence for E-cadherin (A) and N-cadherin (B) in PANC-1 tumor cells. PANC-1 were cultured for 5 days in the absence or presence of 5 ng/ml TGF-β (positive control) or co-cultured with MRC-5 cells (in transwell), then detached and plated again for 24 hours, before fixation for immunodecoration. The nuclei were stained with Hoechst 33342 (blue), cell membranes with anti-CD44 (red in A and B, or yellow in C), and E-cadherin (green in A, or red in B) and N-cadherin (green) with their respective antibodies. The images were acquired under a confocal microscope; 20x lens 2x zoom (A and C), 20x lens (B). Images in A and B represent a single central confocal section; the arrows indicate the points of increased N-cadherin fluorescence in PANC-1 after co-culture with MRC-5. Images in C represent z-stack projections obtained by summing the sections from the basal to the apical cell membrane. In C, the table shows the mean fluorescence intensity (FI) of cadherins' signal, measured on small groups of cells. Mean ± SE ,* $p < 0.05$ (One-way ANOVA). Bar A: 100 μm; bar B and C: 50 μm.

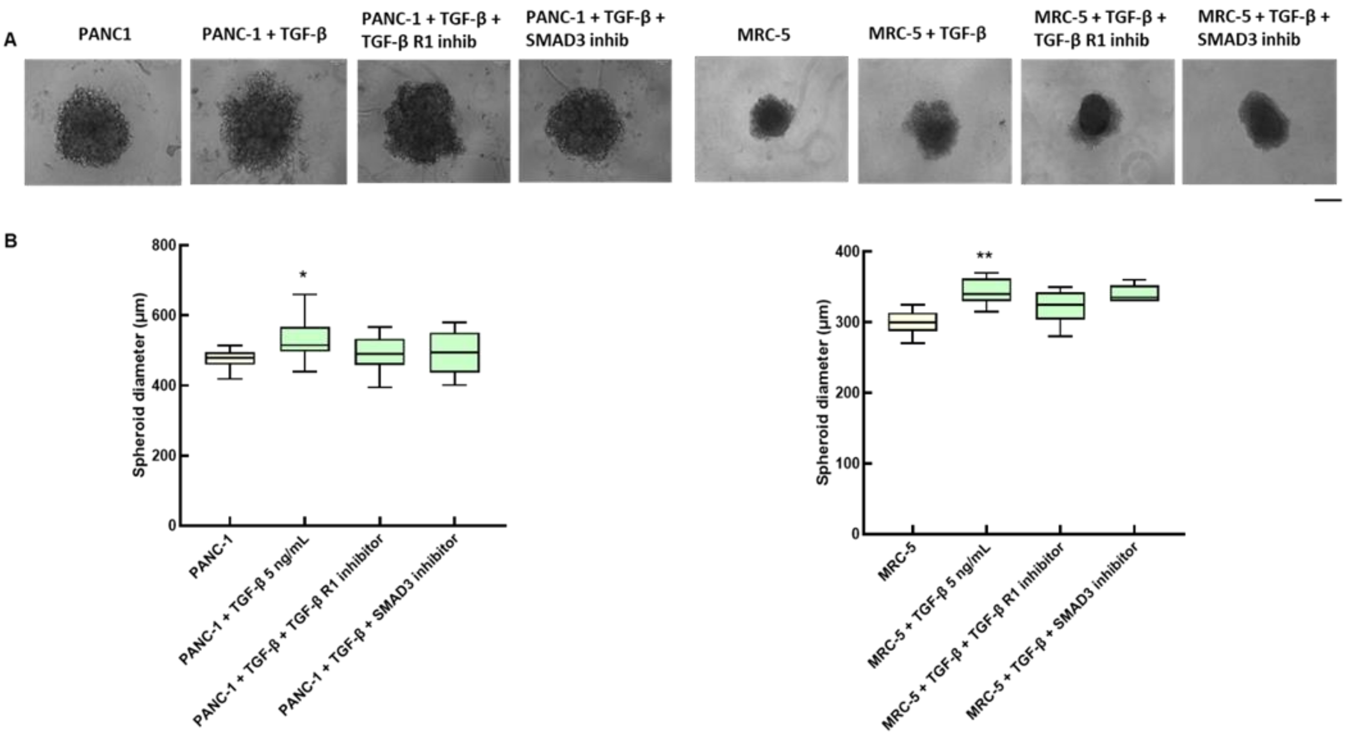

Figure S8. Effect of TGF-β treatment on the dimension of PANC-1 and MRC-5 monospheroids. A) Representative PANC-1 or MRC-5 monospheroids treated with TGF-β (5 ng/mL), with or without 10 µM TGF-β receptor inhibitor (SB431542) or SMAD3 inhibitor (SIS3) 10 µM for 7 days. B) Average diameter of the spheroids, determined using the acquired images (Mean value of 4 different diameters for each spheroid). Data, represented as box-and-whisker plots, are compared by One-way ANOVA. * $p < 0.05$ * (n= 16); ** $p < 0.01$ (n = 6-12). Bar: 200 µm.

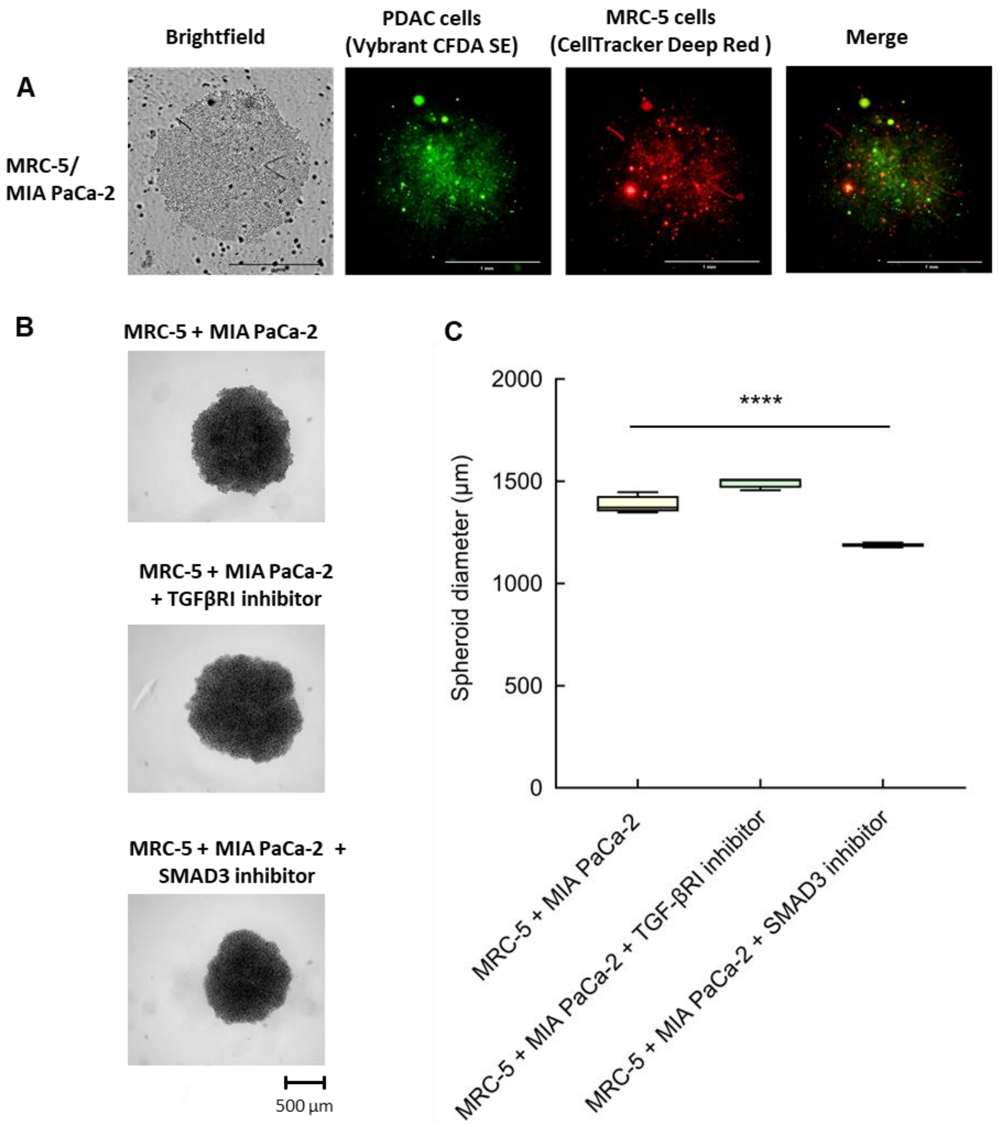

Figure S9. Role of TGF-β on MIA PaCa-2/MRC-5 heterospheroid proliferation. A) Representative heterospheroids made of MIA PaCa-2 cells and MRC-5 cells pre-labelled with Vybrant® CFDA SE and CellTracker™ Deep Red dye, obtained by Operetta CLS Imaging System (5x). Fluorescence images represent a single inner z-stack and were processed in Image J. Scale Bar: 1mm. B) Paula images of MRC-5/PANC-1 heterospheroids treated with 10 µM TGF-βR inhibitor (SB431542), or SMAD3 inhibitor (SIS3) for 7 days. C) Size of the spheroids, determined using the acquired images (average value of 4 different diameters for each spheroid). Data, represented as box-and-whisker plots, are compared by One-way ANOVA. ****p <0.0005 (n = 4).

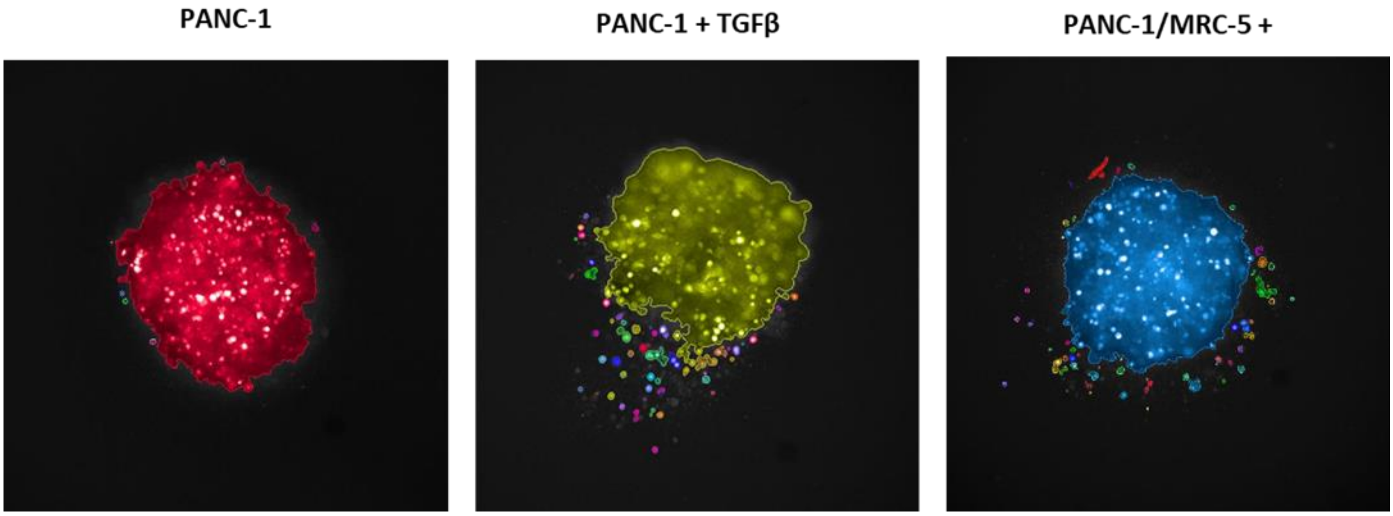

Figure S10. Elaboration by Harmony™ software of images of PANC-1 monospheroids, pre-incubated or not with TGF-β, and PANC-1/MRC-5 heterospheroids obtained by Operetta CLS (5x). Software identifies a large object corresponding to the main spheroid body and other small objects corresponding to spreading cells.

Table S1. TGF-β (pg/mL) released by tumor cells in co-culture and monoculture in the lower chamber (LC) or in the well, respectively, or by monospheroids or tumor cells/MRC-5 heterospheroids into the medium. Mean ± SE are displayed.

	**monoculture (LC/well)**	**co-culture (LC)**	**monospheroid**	**Tumor cells/MRC-5 heterospheroid**
**PANC-1**	618.5 ± 130.9 (8)	603.4 ± 114.2 (8)	1878.8 ± 296.6 (4)	2071.0 ± 542.9 (4)
**MIA-Paca-2**	197.3 ± 50.8 (4)	285.8 ± 37.1 (4)	680.7 ± 64.9 (4)	938.9 ± 107.8 (4)

Disclaimer/Publisher's Note: The statements, opinions and data contained in all publications are solely those of the individual author(s) and contributor(s) and not of MDPI and/or the editor(s). MDPI and/or the editor(s) disclaim responsibility for any injury to people or property resulting from any ideas, methods, instructions or products referred to in the content.
